# *P2rx4* deficiency in mice alleviates allergen-induced airway inflammation

**DOI:** 10.18632/oncotarget.13375

**Published:** 2016-11-15

**Authors:** Andreas Zech, Benjamin Wiesler, Cemil Korcan Ayata, Tilmann Schlaich, Thorsten Dürk, Madelon Hoßfeld, Nicolas Ehrat, Sanja Cicko, Marco Idzko

**Affiliations:** ^1^ Department of Pneumology, University Medical Centre Freiburg, Germany

**Keywords:** asthma, P2RX4, ATP, dendritic cells, IL-1ß, Immunology and Microbiology Section, Immune response, Immunity

## Abstract

Compelling evidences point out a crucial role for extracellular nucleotides such as adenosine triphosphate (ATP) during inflammatory conditions. Once released into the extracellular space, ATP modulates migration, maturation and function of various inflammatory cells via activating of purinergic receptors of the P2Y- and P2X- family. P2RX4 is an ATP-guided ion channel expressed on structural cells such as alveolar epithelial and smooth muscle cells as well as inflammatory cells including macrophages, dendritic cells (DCs) and T cells. P2RX4 has been shown to interact with P2RX7 and promote NLRP3 inflammasome activation. Although P2RX7 has already been implicated in allergic asthma, the role of P2RX4 in airway inflammation has not been elucidated yet. Therefore, we used a selective pharmacological antagonist and genetic ablation to investigate the role of P2RX4 in an ovalbumin (OVA) driven model of allergen-induced airway inflammation (AAI). Both, P2RX4 antagonist 5-BDBD treatment and *P2rx4* deficiency resulted in an alleviated broncho alveolar lavage fluid eosinophilia, peribronchial inflammation, Th2 cytokine production and bronchial hyperresponsiveness. Furthermore, *P2rx4*-deficient bone marrow derived DCs (BMDCs) showed a reduced IL-1ß production in response to ATP accompanied by a decreased *P2rx7* expression and attenuated Th2 priming capacity compared to wild type (WT) BMDCs *in vitro*. Moreover, mice adoptively transferred with *P2rx4*-deficient BMDCs exhibit a diminished AAI *in vivo*. In conclusion our data suggests that P2RX4-signaling contributes to AAI pathogenesis by regulating DC mediated Th2 cell priming via modulating IL-1ß secretion and selective P2RX4-antagonists might be a new therapeutic option for allergic asthma.

## INTRODUCTION

Asthma is one of the world's most common allergic diseases with an increasing prevalence over the last 50 years. Its pathophysiology is characterized by mucus hyper secretion, airway inflammation, variable airflow obstruction associated with bronchial hyper-responsiveness (BHR) to nonspecific stimuli, finally leading to bronchial remodeling. Allergic asthma is induced by allergens such as pollen or house dust mite peptide Derp1 *via* activation of airway epithelia cells, resident dendritic cells (DCs) and macrophages leading to inflammatory cells infiltrating the lung, subsequently initiating a Th2 immune response in the airways [1, 2].

During conditions of ischemia, hypoxia, or inflammation extracellular nucleotides such as adenosine triphosphate (ATP) are released from intracellular storage pools into the extracellular compartment by multiple types of cells [3, 4]. Once in the extracellular space, nucleotides function as damage associated molecular patterns (DAMPs) activating the metabotropic G-protein-coupled P2Y-receptors (P2RY1-14) and the ionotropic P2X-receptors (P2RX1-7) [4, 5]. All P2X-receptors get exclusively activated by ATP, which induces Ca^2+^ influx, causing changes in cell homeostasis [6]. P2RX4 is expressed on cells of the reproductive system [7], in the airways [8-10], on microglia [11] and on cardiac myocytes [12] as well as neutrophils, eosinophils, mast cells, T- and B-lymphocytes [13, 14]. Besides its role in the nervous system, P2RX4 signaling also exerts immunomodulatory functions such as inducing inflammatory-mediated prostaglandin E2 (PGE2) release and facilitating T cell activation at the immune synapse [15, 16]. Furthermore, P2RX4 is involved in the regulation of tracheal smooth muscle cells contraction [8] and modulates surfactant secretion *via* fusion-activated Ca^2+^ entry (FACE) of alveolar type II epithelial cells [9].

Previously we reported that ATP triggers and maintains asthmatic airway inflammation by modulating dendritic cell function [17, 18]. Additionally, local neutralization of ATP abrogates the cardinal features of asthma, including eosinophilic airway inflammation, Th2 cytokine production and BHR in murine models of allergic airway inflammation [5]. Experiments with specific P2R-subtype antagonists and/or knockout animals revealed a contribution of P2RY2, P2RY6, P2RY12 and P2RX7 to allergic airway inflammation [18-21]. Recent studies demonstrated that P2RX4 is co-expressed with P2RX7 in lung epithelial cells and that *P2rx7* deficiency in murine lung epithelial cells is accompanied by an upregulation of P2RX4 as a compensatory mechanism [10]. Additionally, P2RX4 signaling regulates P2RX7 dependent IL-1ß and IL-18 release from bone marrow derived dendritic cells (BMDCs) [22]. Thus, the aim of the study was to elucidate the role of P2RX4 in the pathogenesis of allergic airway inflammation.

## RESULTS

### Increased *P2RX4* expression in human asthmatics and mice with acute airway inflammation (AAI)

**Figure 1:**
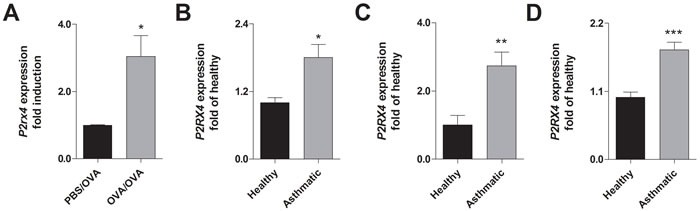
Increased *P2RX4* expression in asthmatic individuals and murine lungs with AAI **A.** Relative *P2rx4* expression in total lung tissue after OVA challenge of OVA-sensitized in comparison to non-sensitized mice. **B.** Relative *P2RX4* mRNA levels in BALF, **C.** blood MNCs and **D.** blood eosinophils of asthmatic individuals compared to healthy controls. *P2RX4* expression was determined using quantitative RealTime-PCR. Graphs show mean ± SD (*n* = 5-8). * *P* < 0.05, ** *P* < 0.01, *** *P* < 0.001 mouse: OVA/OVA *vs* PBS/OVA, human: asthmatic *vs* healthy

In accordance to recent findings [23], mice sensitized to ovalbumin (OVA) and subsequently challenged with OVA-aerosol show an increased *P2rx4* expression in the lung compared to control littermates (Figure 1A). Similar to the results in mice, an elevated *P2RX4* expression could be detected in broncho alveolar lavage fluid (BALF) cells, blood monocytes and eosinophils from asthmatic individuals compared to healthy controls (Figure 1B - 1D).

### Selective blocking of P2RX4 reduces allergen-induced airway inflammation

**Figure 2:**
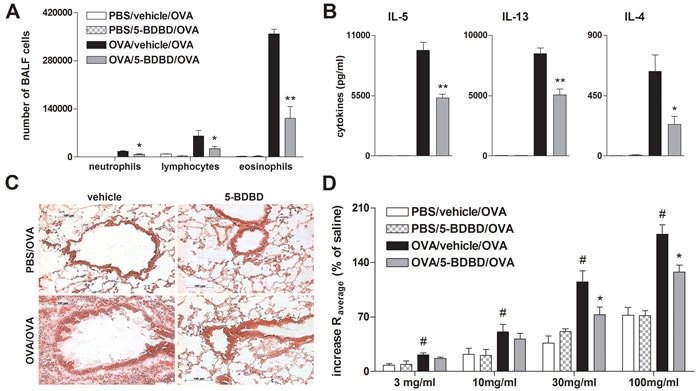
5-BDBD treatment attenuates OVA-induced airway inflammation in mice **A.** BALF differential cell count, **B.** Th2 cytokine concentration in supernatants of OVA re-stimulated MLN cells, **C.** histology of hematoxylin and eosin (H&E) stained lung sections of 5-BDBD and vehicle treated mice (Magnification: 20x objective) and **D.** BHR in response to increasing doses of inhaled methacholine, analyzed by recording the changes in airway resistance [R] and lung compliance [C], after the induction of an airway inflammation using OVA. Graphs show mean ±SEM (*n* = 5-6). * *P* < 0.05, ** *P* < 0.01 OVA/5-BDBD/OVA *vs* OVA/vehicle/OVA. ^#^ *P* < 0.05 OVA/vehicle/OVA *vs* PBS/vehicle/OVA

To investigate the effect of P2RX4-induced signaling on AAI OVA-sensitized mice received the specific P2RX4 antagonist 5-(3-Bromophenyl)-1,3-dihydro-2*H*-benzofuro [3,2-*e*]-1,4-diazepin-2-one (5-BDBD) intratracheally (i.t.) before each of the three consecutive OVA-aerosol challenges. The blockage of P2RX4 reduced the number of BALF eosinophils and lymphocytes (Figure 2A) as well as production of IL-4, IL-5 and IL-13 by mediastinal lymph node (MLN) cells after allergen re-stimulation (Figure 2B). These changes were accompanied by a diminished peribronchial and perivascular inflammation in the lungs (Figure 2C). Consequently, OVA-sensitized 5-BDBD treated animals showed an attenuated bronchial hyperresponsiveness (BHR) in response to increasing doses of inhaled methacholine compared to vehicle treated littermates, determined 24 h after the last OVA challenge by invasive measurement of dynamic resistance and compliance during in the mechanical ventilation (Figure 2D).

### *P2rx4*-deficient mice exhibit attenuated OVA-induced airway inflammation

**Figure 3:**
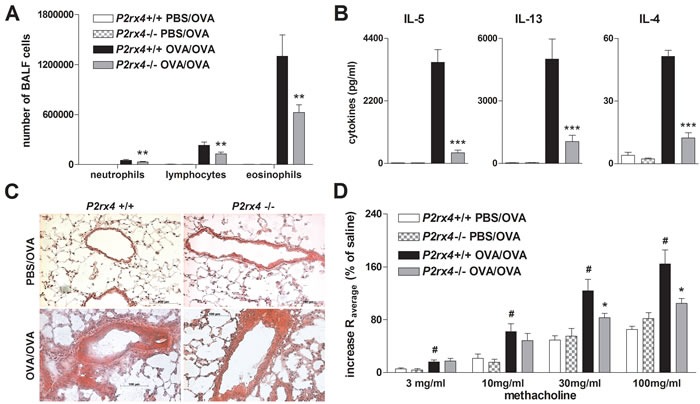
*P2rx4* deficiency is accompanied by a reduction in salient features of experimental AAI **A.** Absolute number of neutrophils, eosinophils and lymphocytes in BALF, **B.** Th2 cytokine content in supernatants of allergen re-stimulated MLN cells and **C.** histology of H&E stained lung sections (Magnification: 20x objective) isolated from WT and *P2rx4* mice after OVA-challenge. **D.** Methacholine mediated BHR represented as changes in airway resistance [R]. Graphs show mean ±SEM (*n* = 5-6). * *P* < 0.05, ** *P* < 0.01, *** *P* < 0.001, *P2rx4* OVA/OVA *vs P2rx4* OVA/OVA. ^#^ *P* < 0.05 OVA/OVA *P2rx4 vs* PBS/OVA *P2rx4*

In order to confirm the antagonist treatment results, we further investigated whether *P2rx4* deficiency is associated with diminished AAI as well. Analogous to 5-BDBD treated mice, *P2rx4*-deficient mice exhibited a reduction in lung infiltrating inflammatory cells including neutrophils, lymphocytes and eosinophils counted in the BALF, in IL-4, IL-5 and IL-13 secretion by OVA re-stimulated MLN cells, in peribronchial inflammation, and in methacholine-induced BHR (Figure 3).

### 5-BDBD alleviates house dust mite extract (HDM) induced allergic airway inflammation

**Figure 4:**
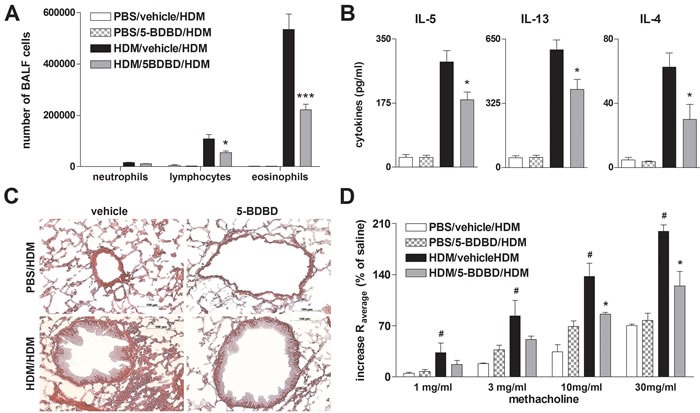
5-BDBD treated mice are partially protected against HDM induced AAI **A.** Differential cell count of BALF, **B.** cytokine concentration in supernatants of HDM re-stimulated MLN cells, **C.** histology of H&E stained lung sections obtained from vehicle and 5-BDBD treated mice after HDM challenge (Magnification: 20x objective). **D.** Changes in airway resistance [R] in response to increasing doses of inhaled methacholine. Graphs show mean± SEM (*n* = 5-6). * *P* < 0.05, ** *P* < 0.01, *** *P* < 0.001, HDM/5-BDBD/HDM *vs* HDM/vehicle/HDM. ^#^ *P* < 0.05 HDM/vehicle/HDM *vs* PBS/vehicle/HDM

Since systemic OVA sensitization and the subsequent OVA aerosol challenges are considered to represent a rather artificial *in vivo* model of AAI, we used a HDM-induced model of eosinophilic airway inflammation to better approach human pathophysiology. The i.t. instillation of HDM extract on day 0, day 7, and day 14 resulted in a strong increase in BALF lymphocytes and eosinophils, lung tissue infiltration, Th2 cytokine release by HDM re-stimulated MLN cells and BHR in response to methacholine in wild type (WT) animals. In line with the results from the OVA-alum model, littermates receiving the P2RX4 antagonist 5-BDBD on day 7 and day 14 before the allergen challenge exhibited an attenuated AAI (Figure 4).

### *P2rx4* deficiency in bone marrow derived dendritic cells (BMDCs) affects ATP-induced IL-1ß secretion, but has no effect on ATP mediated maturation

**Figure 5:**
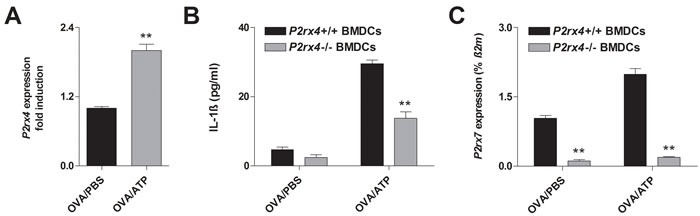
*P2rx4* deficiency in BMDCs is accompanied by a reduced Il-1ß secretion and *P2RX4* expression in response to ATP **A.** *P2rx4* expression in OVA-primed WT BMDCs 24 h after vehicle or ATP (100 μM) stimulation. **B.** IL-1ß release and **C.** *P2rx7* expression of BMDCs isolated from WT and *P2rx4*-deficient mice 24 h after OVA-pulsing together with vehicle or ATP stimulation. P2rx4 and P2rx7 expression graphs are shown as mean ±SD and IL-1ß secretion is represented as mean ±SEM (*n* = 3). * *P* < 0.05, ** *P* < 0.01, *** *P* < 0.001 *P2rx4* expression: OVA/ATP *vs* OVA/PBS. IL-1ß release and *P2rx7* expression *P2rx4*^-/-^ BMDCs *vs P2rx4*^+/+^ BMDCs

Recent reports demonstrated that P2RX4-induced calcium influx is required for P2RX7-dependent production of the pro-inflammatory cytokines IL-1ß and IL-18 by BMDCs [22], suggesting P2RX4-signaling playing a role in dendritic cell function. In fact, adding ATP to cultures of OVA-matured WT BMDCs resulted in an upregulation of *P2rx4* mRNA expression (Figure 5A). Furthermore, OVA-pulsed WT BMDCs showed an increase IL-1ß secretion in response to ATP, while this ATP-induced effect was diminished in OVA-primed *P2rx*4-deficient BMDCs (Figure 5B). Since ATP-mediated NLRP-inflammasome activation and subsequent release of mature IL-1ß are P2RX7-dependent, we determined the *P2rx7* expression in WT and *P2rx4^-/-^* BMDCs. Interestingly, *P2rx7* mRNA levels in OVA-matured *P2rx4-*deficient BMDCs were reduced compared to WT BMDCs and also the upregulated *P2rx7* expression in response to ATP, observed in OVA-primed WT BMDCs, was absent in *P2rx4*^-/-^ OVA-pulsed BMDCs (Figure 5C). Of note, similar to BMDCs LPS activated OVA-primed bone marrow derived macrophages (BMDMs) showed an increased *P2rx4* expression after ATP stimulation and *P2rx4* deficiency was accompanied by an attenuated IL-1ß secretion as well as *P2rx7* expression in response to ATP (Supplementary Figure E1). However, *P2rx4* deficiency did not affect ATP-mediated maturation of OVA-matured BMDCs in terms of CD40, CD80, CD83, CD86 and MHC class II expression (data not shown).

### P2RX4 signaling does not affect ATP-induced migration of DCs

We previously demonstrated that ATP induces DC migration most probably *via* P2RY2 activation [18]. Thus, to investigate whether *P2rx4* deficiency might influence ATP-(P2RY2) induced migration we determined the migration capacity and *P2ry2* expression of *P2rx4*-deficient BMDCs. As demonstrated in supplementary Figure E2, *P2rx4^-/-^* BMDCs and WT BMDCs showed a similar ATP-mediated chemotaxis and *P2ry2* expression.

### *P2rx4*-deficient BMDCs show a reduced Th2 priming capacity *in vitro* and *in vivo*

**Figure 6:**
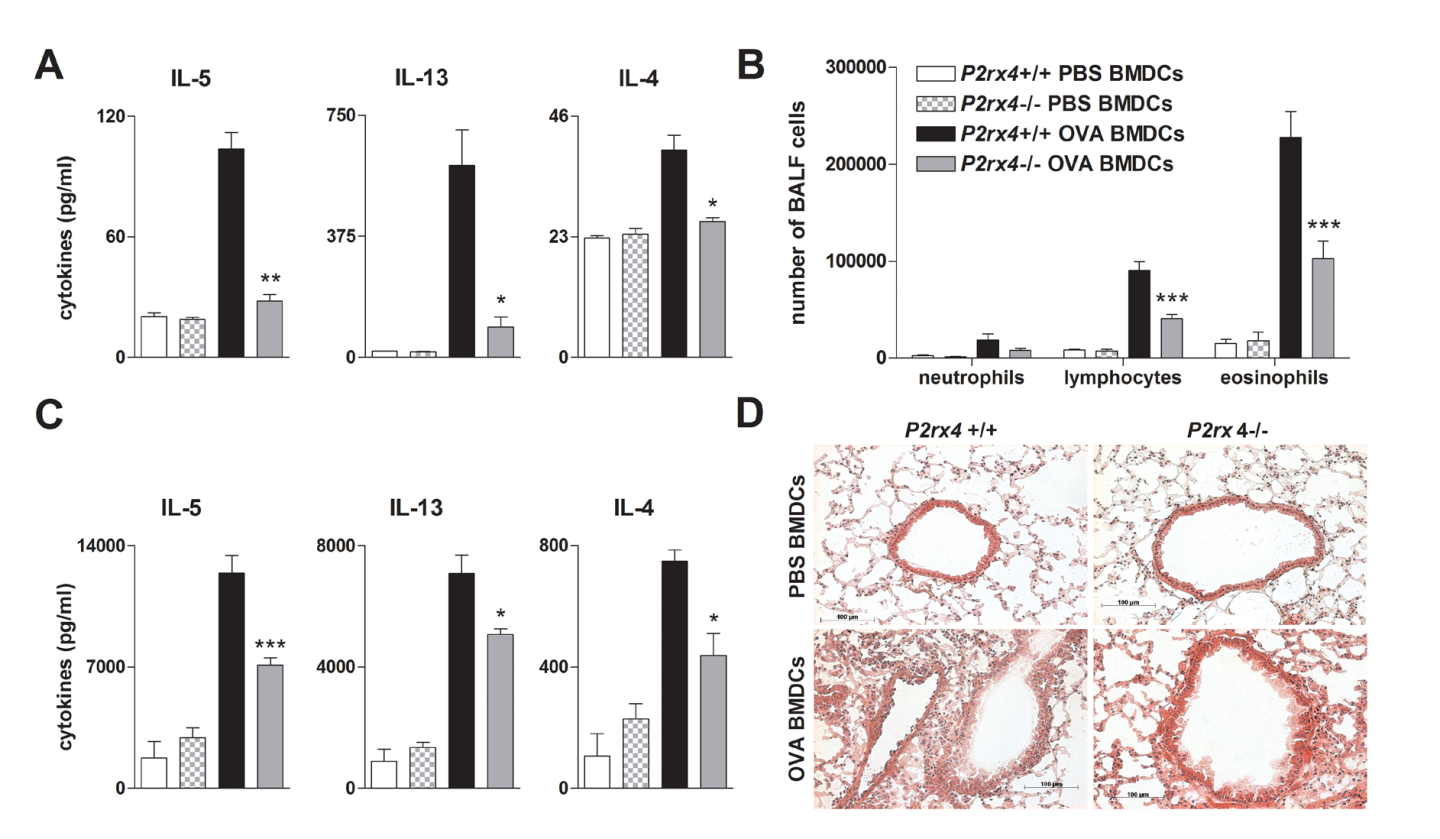
*P2rx4*.deficient BMDCs exhibit an attenuated T-cell priming capacity *in vitro* and *in vivo* **A.** Th2 cytokine secretion of vehicle or OVA-primed WT and *P2rx4*^-/-^ BMDCs cultured together with OVA-specific OTII CD4+ T cells. **B.** BALF differential cell count, **C.** Th2 cytokine levels in supernatants of OVA re-stimulated MLN cells and **D.** histology of H&E stained lung sections isolated from WT mice adoptively transferred with either vehicle- or OVA-primed *P2rx4* or WT BMDCs after OVA-aerosol challenge. Graphs show mean ±SEM (*n* = 3-6). * *P* < 0.05, ** *P* < 0.01, *** *P* < 0.001 *P2rx4* OVA BMDCs *vs P2rx*4+/+ OVA BMDCs.

The ATP-P2RX7 axis has been reported to play a role in Th2-priming. Consequently, blocking P2RX7-signaling in OVA-pulsed BMDCs attenuated Th2-priming capacity [21]. Thus, to investigate the effect of *P2rx4* deficiency in DCs on T cell activation, OVA-primed *P2rx4^-/-^* and WT BMDCs were cultured together with OVA-specific transgenic CD4+ T cells isolated from OTII mice (OTII T cells). In fact, *P2rx4*-deficient OVA-primed BMDCs cultured with CD4+ OTII T cells showed an attenuated Th2-priming capacity in terms of IL-4, -5 and -13 secretion compared to co-cultures including OVA-pulsed WT BMDCs (Figure 6).

To address the relevance of *P2rx4* deficiency on DC function *in vivo*, we used a DC driven model of AAI. Therefor *P2rx4^-/-^* and WT BMDCs were primed with OVA or PBS overnight and subsequently adoptively transferred into WT mice *via* i.t. administration. As expected, WT mice receiving *P2rx4*-deficient OVA-pulsed BMDCs showed less lymphocytes and eosinophils in the BALF, a diminished Th2 cytokine release by OVA re-stimulated MLN cells as well as a reduced peribronchial inflammation after OVA-challenge compared to littermates receiving OVA-pulsed WT BMDCs (Figure 6B, 6C and 6D).

## DISCUSSION

During the last decade extracellular purines and the purinergic receptors emerged as important components of the immune system due to their capability to initiate and modulate immune responses. Although purinergic signaling plays a crucial role in host defense against bacterial infections, its dysregulation is linked to pathogenesis of chronic inflammatory diseases including allergic asthma [5]. Extracellular ATP exerts multiple immune modulatory effects such as maturation, migration and the induction of cytokine/chemokine release *via* binding to ionotropic P2X- as well as metabotropic P2Y-receptors, expect P2RY6 and P2RY14, in an autocrine or paracrine manner [5, 14]. We previously demonstrated that ATP-induced P2RY2 and P2RX7 signaling play an important role in AAI in mice and man [18, 21]. However, also functional interactions and co-expression of P2RX7 and P2RX4 on lung epithelial cell, macrophages and DCs have been demonstrated recently [10, 22, 24], the effect of P2RX4-signaling on AAI pathogenesis is not completely elucidated yet.

In the present study, we demonstrate that mice with experimental allergic airway inflammation and human allergic asthmatics show an elevated *P2RX4* expression compared to healthy individuals. This is of functional relevance for asthma pathogenesis since selective inhibition of P2RX4 using 5-BDBD alleviates cardinal features of AAI, including airway eosinophilia, peribronchial inflammation, Th2 cytokine secretion of allergen re-stimulated MLN cells and BHR to increasing doses of inhaled methacholine in the murine model of OVA-induced airway inflammation. In addition, in mice with an HDM-induced airway inflammation, better resembling human condition, the administration of 5-BDBD also results in an attenuated disease phenotype. Observing similar results in *P2rx4*-deficient mice further validated the contribution of P2RX4 to AAI.

P2RX4 has been associated with ATP-induced NLRP3-inflammasome activation and subsequent release of mature IL-1ß in a P2RX7-dependent manner [22, 25]. IL-1ß is a strong enhancer of inflammation showing an increased expression in the airways of asthmatic individuals, therefore most likely contributing to asthma pathogenesis [26, 27]. Several studies showed that IL-1ß is required for Th2 cell activation and consequently blocking IL-1ß signaling attenuates OVA-induced airway inflammation [28-30]. Additionally, a perturbed IL-1ß production of DCs observed in *P2rx7*-deficient mice has been associated with an attenuated AAI as well [21]. Here we demonstrate that OVA-primed BMDCs exhibit an increased *P2rx4* and *P2rx7* expression as well as an elevated IL-1ß secretion in response to ATP. Thereby, the depletion of P2RX4 results in a diminished ATP-mediated IL-1ß release. Interestingly, *P2rx4*-deficient OVA-primed BMDCs show decreased *P2rx7* expression in both, vehicle and ATP stimulated condition, suggesting P2RX4 signaling to modulate *P2rx7* expression. In accordance with our findings a decreased *P2rx7* expression in various tissues including liver, spleen and peritoneal macrophages of *P2rx4*-deficient mice has been reported previously [31, 32]. In contrast, Weinhold et al. observed a compensatory up-regulation of P2RX7 in an airway epithelial cell line as a result of knocking down *P2rx4* [10]. Nevertheless, in addition to BMDCs LPS-activated BMDMs showed an elevated *P2rx4* expression after OVA-priming and additional ATP-stimulation. Furthermore, similar to BMDCs, *P2rx4* deficiency in LPS-activated OVA-pulsed BMDMs is accompanied by a diminished IL-1ß secretion and *P2rx7* expression in response to ATP.

Myeloid DCs are professional antigen processing and presenting cells playing a crucial role in initiating as well as maintaining AAI [17]. Thereby, purinergic signaling has been shown to modulate DC migration, maturation and function [17, 21]. The ATP-mediated activation of P2RY2 facilitates DC migration *in vitro* and DC lung recruitment *in vivo* [18]. Here, we demonstrate that WT and *P2rx4*-deficient BMDCs exhibit a comparable chemotactic capacity in response to ATP *in vitro*, which correlates with the similar *P2ry2* expression levels measured in both BMDC populations. In addition, like previously reported for *P2rx7*-deficient BMDCs [21], *P2rx4* deficiency did not affect ATP-mediated BMDC maturation. DCs priming naïve T cells to the allergen-specific Th2 phenotype is a pivotal step during allergen sensitization and a prerequisite for allergic asthma pathogenesis in mice [33, 34]. In this study, we reveal that OVA-pulsed *P2rx4*-deficient BMDCs cultured together with OTII CD4+ T cells show an attenuated Th2 priming capacity by means of IL-5, IL-13 and IL-4 production. This observation is of *in vivo* relevance, since adoptive transfer of *P2xr4*-deficient OVA-primed BMDCs into WT mice prior to OVA challenge resulted in reduced lymphocyte and eosinophil counts in the BALF, Th2 cytokine production of OVA re-stimulated MLN cells and peribronchial inflammation compared to littermates adoptively transferred with OVA-pulsed WT BMDCs.

In summary, we report that P2RX4 signaling contributes to the pathogenesis of OVA-induced AAI *via* promoting IL-1ß secretion and Th2 priming of dendritic cells. Thus P2RX4 plays an important role during the intiation of allergen-induced inflammation by driving Th2 immunity. Moreover, our observation that P2RX4 ablation decreases *P2rx7* mRNA expression in OVA-primed BMDCs, especially after ATP stimulation, is consistent with the correlation P2RX4 and P2RX7 reported before [24, 32]. Thus one could hypothesize that the decreased AAI in *P2rx4*-deficient mice is due to the alleviated *P2rx7* expression and signaling observed in these animals. However, our data also demonstrates that selective pharmacological blocking of P2RX4 in WT mice results in diminished AAI as well, which provides convincing evidence for a functional relevance of P2RX4 signaling in asthma pathogenesis rather than a phenotype exclusively caused by the downregulated *P2rx7* expression. Accordingly, a role of P2RX4 in airway remodeling by regulating ciliary beat, alveolar fluid transport and surfactant secretion of airway epithelial cells has been emphasized previously [35, 36]. Taken together, targeting P2RX4 might provide a potential promising therapy for allergic airway inflammation.

## MATERIALS AND METHODS

### Mice

Balb/c, C57BL/6 mice and B6.Cg-Tg(TcraTcrb)425Cbn/J (OTII) were purchased from Charles River (Sulzfeld, Germany), *P2rx4*-deficienct C57BL/6 mice were designed in the lab of Francois Rassendren as previously described [37] and bred under specific pathogen free (SPF) conditions at the animal facility of Freiburg University. All mouse experiments were performed in accordance to the local animal ethic committee (G12-095; G12-096).

### OVA-alum induced AAI

The OVA-alum model was performed as previously described [18]. Briefly, mice were sensitized by an intraperitoneal (i.p.) injection of OVA grade V (Worthington Biochemical Corp., Lakewood, NJ)/alum (Thermo Scientific, Waltham, MA) on days 0 and 7, and were challenged with 1 % OVA aerosols (Sigma Aldrich, Taufkirchen, Germany) on three consecutive days, starting on day 17. Thirty minutes before each allergen challenge, animals were anesthetized using ketamine and xylazine, and given an i.t. injection of 80 μl control vehicle or 80 μl 100 μM P2RX4 antagonist 5-(3-Bromophenyl)-1,3-dihydro-2H-benzofuro [3,2-e]-1,4-diazepin-2-one (5-BDBD, Tocris, Bristol, UK). On day 20 mice were either anesthetized for lung function measurement or sacrificed for BALF collection followed by lung resection and storage in OCT freezing medium (Sakura Finetek Europe B.V, Alphen aan den Rijn, Netherlands) 24 hours after the last OVA exposure. Isolated mediastinal lymph node (MLN) cells were cultured in round bottom 96 well plates (2x10^5^ cells/well) in 200 μl RPMI medium (gibco life technologies, Carlsbad, CA) and re-stimulated with OVA (Worthington Biochemical Corp.) for 5 days before collecting the supernatants. IL-4, IL-5, and IL-13 concentrations were determined by enzyme-linked immune-sorbent assay (ELISA) using R&D DuoSets (R&D Systems, Minneapolis, MN).

### Flow cytometry

Fluorescence-activated cell sorting (FACS) was performed as previously described [38]. Briefly, BALF cells were incubated with an unlabeled anti-CD16/CD32 antibody for blocking Fc receptors to avoid unspecific binding before using anti-Gr-1 FITC-conjugated, -CCR3 PE-conjugated, -CD3 and -B220, both Cy7-conjugated and -CD11c APC-conjugated antibodies (eBioscience, SanDiego, CA) for FACS analysis. Flow cytometry was performed using a FacsCalibur flow cytometer (BD Biosciences, San Jose, CA) and data was analyzed using FlowJo software (TreeStar, Ashland, OR).

### Invasive lung function measurement

Deeply anesthetized mice were intubated with an 18-gauge catheter, placed in a plethysmograph (EMMS, Bordon, UK) and mechanically ventilated (Minivent 485, Hugo-Sachs, March, Germany). Respiratory rate was set at 200 breaths per minute with a tidal volume of 200 μl and a positive end expiratory pressure of 2 ml H2O. Mice received increasing concentrations of nebulized methacholine (0 mg/ml, 3 mg/ml, 10 mg/ml, 30 mg/ml, 100 mg/ml) while resistance and compliance were recorded by a trans-pulmonary pressure transducer (TPP 200, EMMS, Bordon, UK) connected to an amplifier interface unit (AAC 091, EMMS, Bordon, UK) using eDacq software (EMMS, Bordon, UK).

### Generation of BMDCs

Bone marrow cells were isolated from tibia and femur by flushing the medullary cavity. 2x10^6^ BM cells were cultivated in 2 ml RPMI medium, containing 10 % fetal calf serum (FCS, Biocell Laboratories, Rancho Dominguez, CA), 1 % penicillin/streptomycin, β-Mercaptoethanol and murine granulocyte macrophage colony-stimulating factor (GM-CSF, 200 IU/ml, ImmunoTools, Friesoythe, Germany) in a 6-well plate. Additional 2 ml medium were added on day 3 and medium was exchanged completely on day 6. On day 7 the purity of BMDCs was greater than 90 % as determined by FACS. BMDCs were stimulated with OVA (200 μg/ml) and ATP (100 μM, Sigma Aldrich), used for co-culture with OVA-specific TCR transgenic CD4+ T cells (OTII) or adoptively transferred.

### BMDCs and OTII cell co-culture

2x10^4^ OVA-primed (100 μg/ml, Worthington Biochemical Corp.) BMDCs were cultured together with 1x10^5^ OTII CD4+ T cells in a U-shape 96-well plate. Supernatants were collected after 72 h and 120 h for determination of Th2 cytokine concentration (IL-4, -5, -13) using ELISA.

### Induction of airway inflammation by i.t. application of OVA-primed BMDCs

BMDCs were pulsed with 100 μg/ml OVA (Worthington Biochemical Corp.) or vehicle overnight. 1x10^6^ OVA- or vehicle-primed BMDCs were adoptively transferred into target mouse *via* i.t. instillation. Ten days after transfer, mice were exposed to aerosolized OVA (Sigma) for three consecutive days and sacrificed 24 h after last OVA exposure.

### Quantitative PCR

Quantitative PCR was performed on the LightCyler 480 (Roche, Mannheim, Germany) using the Takyon mastermix, (Eurogentec, Köln, Germany). For all reactions an annealing temperature of 60°C has been used. Primers and dual labeled probes were designed as previously described [39], sequences are available upon request. ß2m or GAPDH were used as reference gene (RG). Percent RG values were calculated using the formula: %RG = 100 x 2^(-Ct)^. Combined standard deviations of reference gene and gene of interest (GOI) were calculated using the formula: SD = 100 x 2^(-DCt)^ x ((ln2 x SD_RG_)^2^ + (ln2 x SD_GOI_)^2^)^1/2^ [40].

### Statistical analysis

The statistical significance of differences between groups was calculated using one-way ANOVA, followed by Bonferoni comparison test or using *t* test for comparing two single values. Differences were considered significant at *P* < 0.05.

## SUPPLEMENTARY MATERIALS METHODS



## Abbreviations

AAI: Acute airway inflammation
ATP: Adenosine triphosphate
BALF: Broncho-alveolar-lavage-fluid
5-BDBD: 5-(3-Bromophenyl)-1,3-Dihydro-2*H*-Benzofuro [3,2-*e*]-1,4-Diazepin-2-one
BHR: Bronchial hyper responsiveness
BM: Bone marrow
BMDCs: Bone marrow derived dendrtitic cells
BMDM: Bone marrow derived macrophages
DAMPs: Damage-associated molecular patterns
DCs: Dendritic cells
FACE: Fusion-activated Ca2+ entry
FACS: Fluorescence-activated cell sorting
GM-CSF: Granulocyte macrophage colony-stimulating factor
HDM: House dust mite
H&E: Hematoxylin and eosin
IL: Interleukin
i.p.: Intraperitoneal
i.t: Intratracheally
MLN: Mediastinal lymph node
OTll T cells: OVA-specific T-cell-receptor transgenic CD4+ cells
OVA: Ovalbumin
P2R: Purinergic-type 2-receptor
PGE2: Prostaglandin E2
SPF: Specific Pathogen Free
Th2: T-helper cell type 2
WT: Wild type
