# Supplementary material for: P2rx4 deficiency in mice alleviates allergen-induced airway inflammation
Source: Oncotarget. 2016 Nov 15;7(49):80288–97. doi: 10.18632/oncotarget.13375 (PMC5348320; doi:10.18632/oncotarget.13375)
Supplement: Supplementary file 1 [file oncotarget-07-80288-s001.pdf]

P2rx4 deficiency in mice alleviates allergen-induced airway inflammation

Supplementary Methods

Murine model of house dust mite extract (HDM) induced airway inflammation

For sensitization 100 µg of HDM *(Dermatophagoides pteronyssinus,* Greer Laboratories, London, UK) was administered intratracheally to deeply anesthetized mice on day 0 and 7. I.t. instillation of HDM was repeated on day 14 as challenge. 72 h after the last HDM application mice were either sacrificed to obtain BALF cells for FACS analysis, lung sections for histology and mediastinal lymph node cells for allergen re-stimulation to determine Th2 cytokine production, or anesthetized for bronchoalveolar hyperresponsiveness measurement. Control mice received vehicle (PBS) on day 0 and 7 instead of HDM.

BMDC *in vitro* migration assay

Triplicates of 1×10^5 BMDCs in 100 µl RPMI medium without supplements (Gibco Life Technologies, Carlsbad, CA) were put in a 5 µm transwell insert placed in a 24-well plate (Corning, Amsterdam, Netherlands). Lower compartments were loaded with increasing concentrations of ATP. The plate was incubated at 37°C with 5 % CO_2 for 2 hours. Chemotactic index was calculated by dividing number of cells from each lower compartment by the number of migrated cells in medium control.

Generation of bone marrow derived macrophages (BMDM)

The murine femur and tibia were flushed with DMEM medium containing 10 mM L-Glutamin, 10 % FCS (Biocell Laboratories, Rancho Dominguez, CA), and 1 % penicillin/streptomycin to obtain bone marrow cells. 4×10^6 bone marrow cells were incubated at 37°C with 5 % CO_2 in 10 ml DMEM medium supplemented with 100

IU/ml recombinant murine macrophage colony-stimulating factor (M-CSF, Immunotools, Friesoythe, Germany). Additional 5 ml medium supplemented with M-CSF were added on day 3. On day 7 BMDMs were collected and plated in a 24-well plate using $5x10^5$ cells per well. BMDMs were activated by adding LPS (3 µg/ml) for 6 h.

Supplementary table

No.	Sex	Age	FEV_1 (%pred)	Medication	Total IgE [kU/l]	Allergen specific IgE [kU/l]	Allergen
AP							
1*	m	32	83	BA, IC	352	77.1	DP
2*	f	27	102	BA	226	14.0	DP
3*	f	22	98	BA, IC	1820	32.1	DP
4*	m	24	104	BA, IC	195	53.1	DP
5*	f	26	79	BA	450	23.0	DP
6	f	28	95	BA, IC	345	14.6	DP
7	m	21	92	BA, IC	1320	45.7	Birch
8*	f	22	89	BA, IC, CR	280	59.7	Birch
HC							
1*	f	30	97		91		
2*	m	27	107		93		
3*	m	28	94		69		
4*	f	27	92		8		
5*	m	25	114		56		
6*	m	28	112		37		
7	f	24	102		27		
8	m	27	97		20		

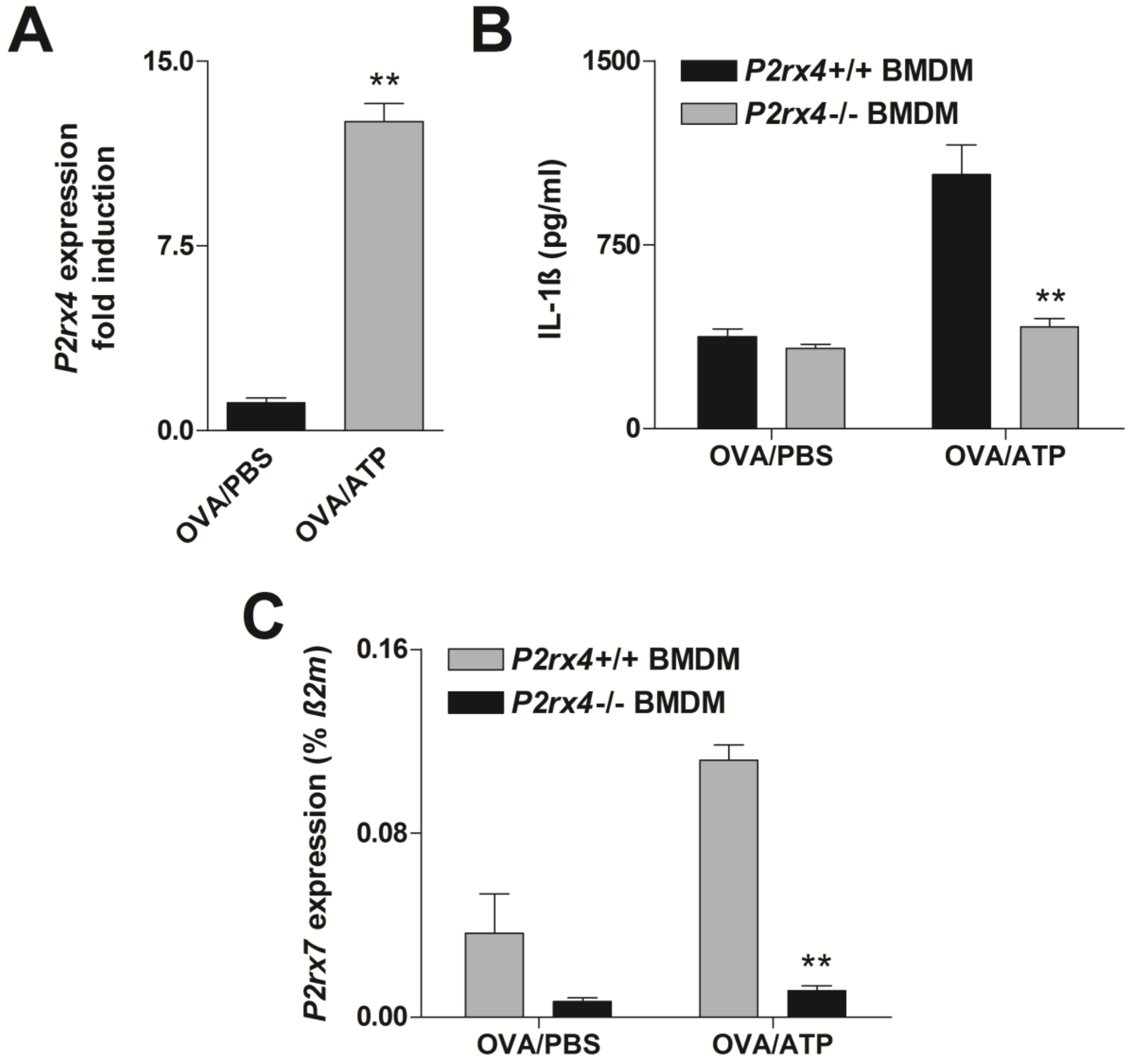

Supplementary Figure E1

(A) *P2rx4* expression in OVA-primed bone marrow derived macrophages (BMDMs) after vehicle or ATP (1 mM) stimulation. (B) IL-1ß release and (C) *P2rx7* expression of BMDMs isolated from WT and *P2rx4*-deficient mice 24h after PBS/PBS, OVA/PBS or OVA/ATP stimulation. The qPCR results are represented as mean ±SD and the IL-1ß graph shows mean ±SEM of one experiment out of 3. ** $P < 0.01$ *P2rx4*$^{-/-}$ vs *P2rx4*$^{+/+}$. *P2rx7* Expression: *P2rx4*$^{-/-}$ vs *P2rx4*$^{+/+}$

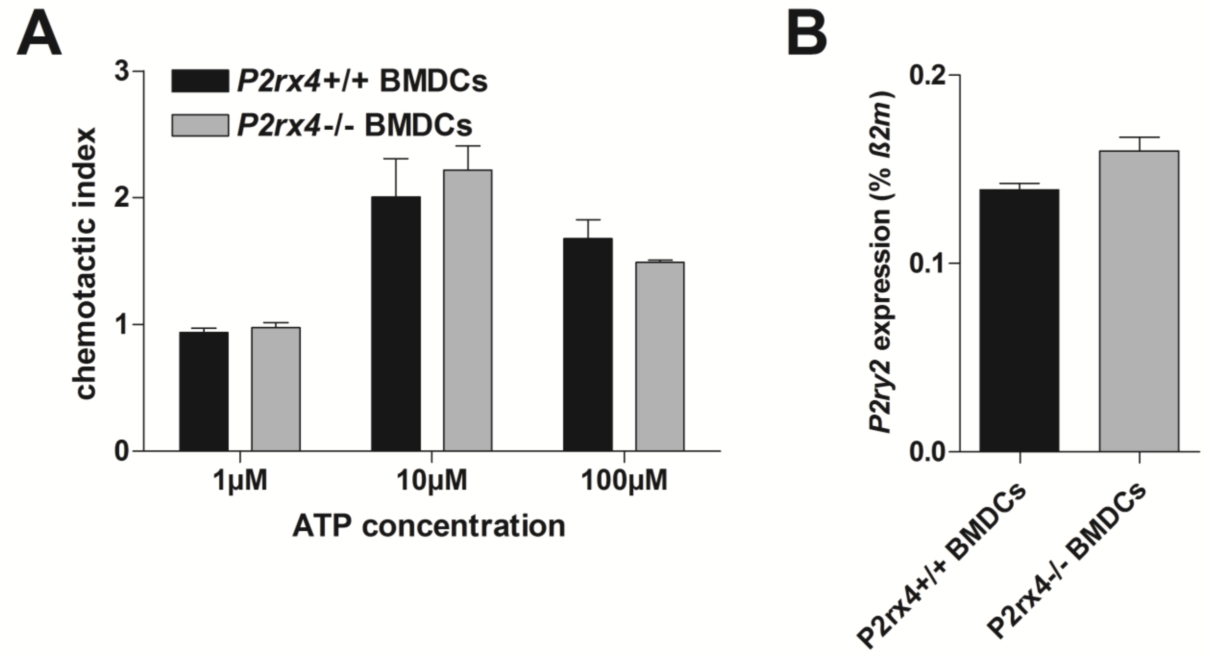

Supplementary Figure E2

(A) *In vitro* chemotaxis of *P2rx4*$^{+/+}$ and *P2rx4*$^{-/-}$ bone marrow derived dendritic cells (BMDCs) towards different concentrations of ATP relative to PBS control. (B) Expression of *P2ry2* in OVA-primed BMDCs stimulated with vehicle or ATP (100 µM). Migration graph shows mean ±SEM and *P2ry2* expression is represented as mean ±SD of one experiment out of 3.
